# Five-Level Anterior Cervical Discectomy And Fusion

**DOI:** 10.7759/cureus.19961

**Published:** 2021-11-28

**Authors:** Denis Babici, Phillip M Johansen, Timothy D Miller, Brian Snelling

**Affiliations:** 1 Neurology, Charles E. Schmidt College of Medicine, Boca Raton, USA; 2 Neurological Surgery, Charles E. Schmidt College of Medicine, Boca Raton, USA; 3 Neurosurgery, Boca Raton Regional Hospital, Boca Raton, USA

**Keywords:** x-ray analysis, intraoperative fluoroscopy, craniocervical fusion, high-resolution ct scan, anterior cervical corpectomy

## Abstract

Anterior cervical discectomy and fusion (ACDF) is a common treatment modality that has shown good clinical results in patients with cervical degenerative disc disease. ACDF remains the procedure of choice for most patients given its satisfactory clinical outcomes and proven radiological fusion ranging from 90-100%. Five-level ACDF is a very rare type of surgery, even in large spine centers. This type of procedure is unique because, beyond three or four levels, the surgeon needs to switch from a transverse incision to a longitudinal incision along the medial sternocleidomastoid (SCM) muscle border, which is less preferred for cosmetic reasons. Another reason why this procedure is seldom performed is that extreme multilevel ACDF is associated with higher complication and failure rates. Literature covers one, two, and three-level anterior surgeries, but there are few studies reporting the outcomes of five-level ACDF. In the few studies that do report five-level ACDF, the data is controversial. Some studies show the risk of adjacent-segment disease increasing with a higher number of fused levels and increasing incidences of reoperation. Other studies show no changes in the risk of adjacent segment disease in multilevel ACDF in comparison with single-level ACDF. One study even showed a decreased level of adjacent-segment disease and reoperation rates in multilevel ACDF when compared to single-level ACDF. To contribute to current knowledge, we share our experience with five-level ACDF. We report the case of a 63-year-old female who presented with complaints of progressively worsening weakness in the upper extremities. MRI of her cervical spine demonstrated multilevel degenerative disc disease throughout C3-T1 with reversal of normal lordosis and a kyphotic deformity. We performed a successful ACDF at C3-T1 as well as partial corpectomy of the C5 and C6 vertebrae. We did it through a standard transverse incision from the midline to the medial border of the SCM within a preexisting neck crease, demonstrating that in select patients, extreme multilevel ACDF can be performed with proper anatomical dissection and without the need for multiple or longitudinal incisions.

## Introduction

Anterior cervical discectomy and fusion (ACDF) are one of the more common procedures for treating degenerative disc disease of the cervical vertebrae. The “discectomy” refers to the removal of the intervertebral disc, including the herniated portion, to provide decompression of the spinal cord. The “fusion” refers to the additional surgical procedure to stabilize the two adjacent vertebrae, which, theoretically, will be compromised after removing the intervertebral disc [1]. Nationally, approximately 132,000 ACDFs are done each year [2]. Five-level ACDF is a very rare type of surgery, even in large spine centers. Literature covers one, two, and three-level anterior surgeries, but the current literature is lacking in studies reporting the outcomes of five-level ACDF [3]. This type of procedure is rare because, beyond three or four levels, the surgeon must switch from a transverse to a longitudinal incision along the medial border of the sternocleidomastoid (SCM) or utilize multiple transverse incisions [4]. This incision, however, is less favorable for cosmetic reasons compared to a transverse incision that can be performed within an existing skin crease. Another reason why this procedure is seldom performed is because extreme multilevel ACDF is associated with higher complication and failure rates [3]. We report a case of five-level ACDF for two reasons: 1) the paucity of available literature regarding extreme multilevel ACDF 2) demonstration that, in select patients, extreme multilevel ACDF can be performed with proper anatomical dissection utilizing a standard transverse incision and without the need for multiple transverse or longitudinal incisions.

## Case presentation

**Figure 1:**
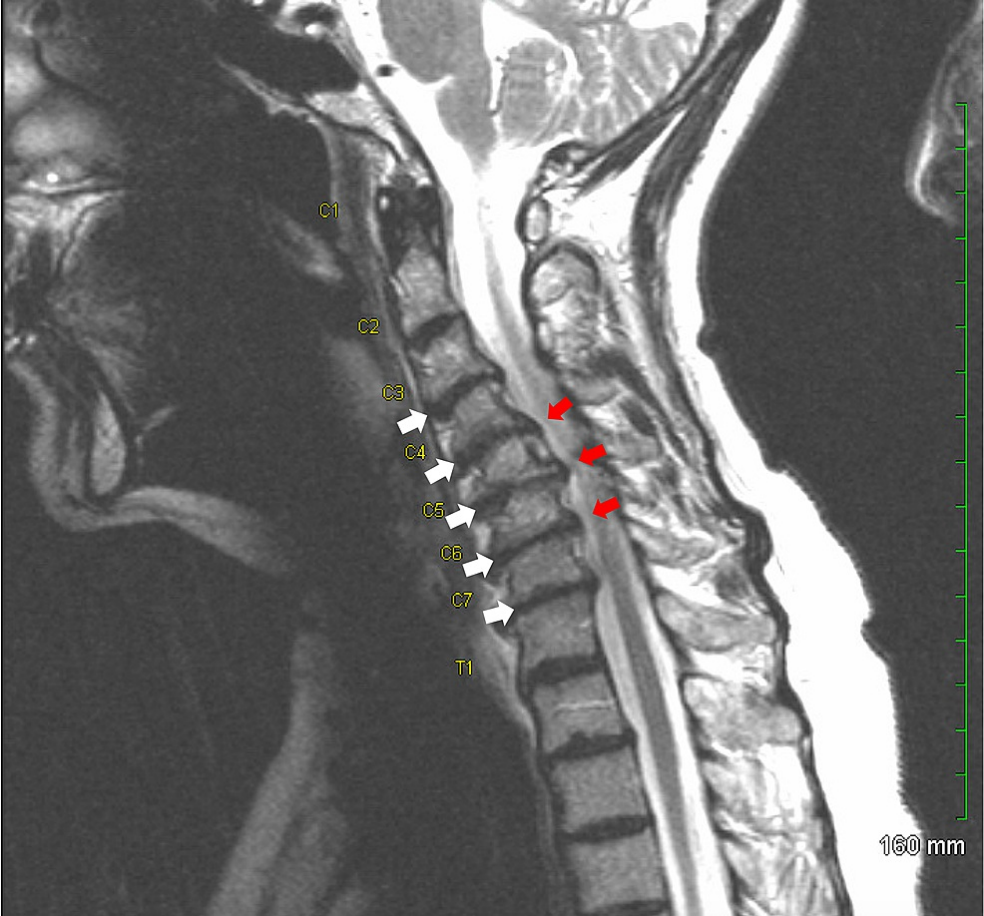
Sagittal view MRI of the cervical spine Multilevel cervical degenerative disc disease with loss of normal cervical lordosis (white arrows) Levels of mild to severe central stenosis from C4 to T1 (red arrows)

A 63-year-old female with no past medical or surgical history presented to the clinic with complaints of progressively worsening weakness and pain in the upper extremities bilaterally. MRI of the cervical spine demonstrated multilevel cervical degenerative disc disease with reversal of normal lordosis and a kyphotic deformity in addition to varying levels of moderate to severe central canal stenosis from C4-T1 and moderate to severe bilateral foraminal stenosis at almost every level, especially on the right side (Figure 1).

We discussed the various conservative and surgical options with the patient, and it was determined that surgery would provide the best chance to stop the progression of her neurological symptoms. Due to her cervical kyphosis, we opted for an anterior approach to restore vertebral column height and lordotic curvature. The anterior fusion would supplement with posterior instrumentation given the number of levels requiring fusion. 

Description of the surgery

**Figure 2:**
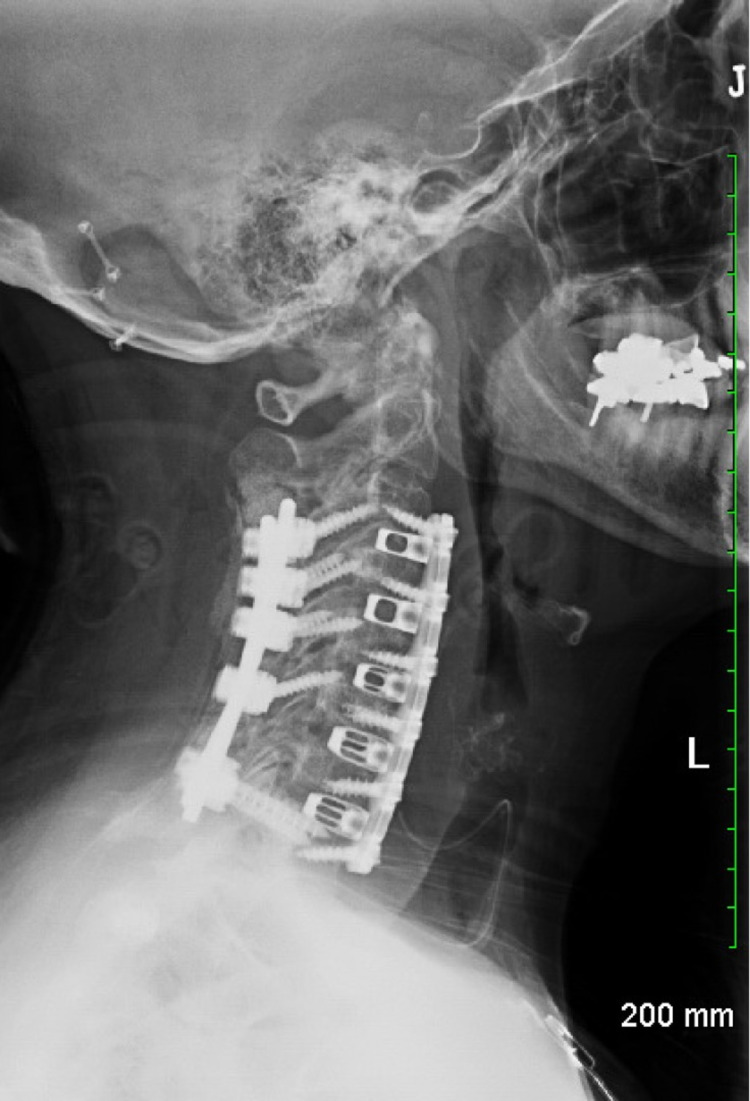
Lateral X-ray of the cervical spine Proper placement of all hardware

The left-sided approach was chosen to theoretically decrease the risk of recurrent laryngeal nerve injury. A transverse incision was created extending from medical sternocleidomastoid to midline in existing neck crease, planned using fluoroscopic localization overlying the C5-6 interspace. Standard neck dissection was performed. Omohyoid muscle was divided sharply (and reapproximated at the end of the case). Discectomy and graft placement proceeded superiorly to inferiorly with Caspar pin retractors for retraction two levels at a time, C3-5, C5-7, then C7-T1. The posterior osteophyte complexes were removed to decompress the spinal cord and exiting nerve roots. Six-degree lordotic cages were used at each level to restore natural cervical lordosis. Following the anterior portion of the case, the patient was turned prone on a Jackson frame, and her head was immobilized in a neutral position with a Mayfield head holder. Subsequently, posterolateral screw and rod instrumentation in a standard fashion was placed utilizing stereotactic navigation with lateral mass screws from C3-6 and pedicle screws at T1 (Figure 2).

**Figure 3:**
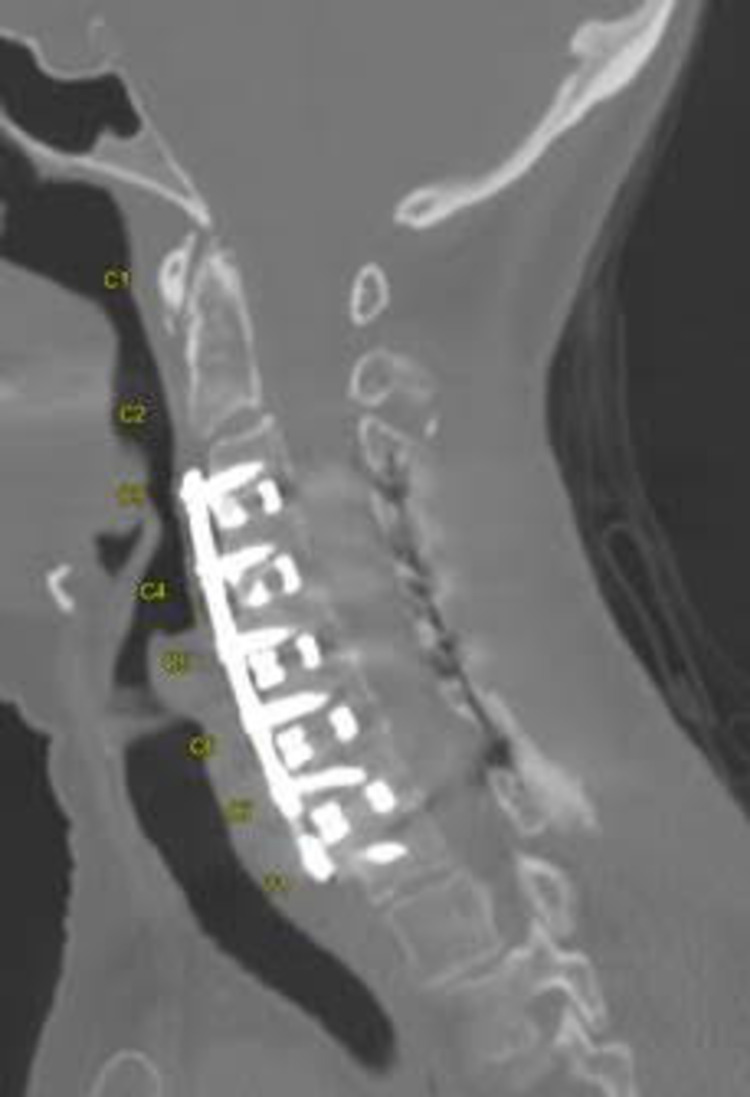
Midline sagittal CT scan of the cervical spine nine weeks after surgery Good bone healing without any screw lucency

The patient tolerated the procedure without complication, was discharged to the rehabilitation center on day three. She followed up at nine weeks, at which time a CT scan of the cervical spine was performed showing good bony fusion (Figure 3), and her hard cervical collar was discontinued. At nine weeks post-surgery, her symptoms have significantly improved.

## Discussion

Multilevel discectomies and corpectomies are often required for the cure of degenerative disorders, post-traumatic or post-surgical deformities, and neoplasia-related instabilities. In these situations, diffuse spinal canal narrowing with ventral spinal cord compression and kyphosis are common, and the only realistic surgical options use an anterior approach [3]. In the literature, the clinical results of multilevel cervical anterior fusion constructs vary, and only a few studies focus on the clinical and geometrical outcomes of four- and five-level fusions. Unfortunately, details on the number of instrumented vertebrae and distribution of decompressed vertebrae are often lacking. Anecdotally, construct failures in multilevel corpectomies with stand-alone strut grafts have been reported and reviewed as high as 10-50% [3,5,6]. Using plates with rigid screw-plate locking mechanisms reports on graft, cage, and plate failures, particularly in multilevel corpectomies, raised concerns on the limitations of these devices [7]. Failures increased as the number of decompressed levels increased [3,8,9]. Regarding the long-term performance following anterior five- and six-level decompressive surgeries, it remains an open question if reconstruction of ‘normal’ cervical lordosis is imperative or if there is an alternate, optimal degree of lordosis. The literature offers some hints that reconstruction of cervical lordosis might be favorable concerning the clinical outcomes and neurologic recovery [10]. One study showed that anterior-only instrumentation following segmental decompressions, or the use of hybrid techniques with discontinuous corpectomies, can bypass the need for posterior supplemental surgery in four- and five-level surgeries [3]. The five-level ACDF is a very rare procedure, and in the literature, there is controversy regarding complication rates of multilevel ACDF. For example, Dang et al. used a finite element model to show that mechanical load strain in the adjacent segment is much higher after two-level fusion than after one-level fusion [11]. Clinically, the number of fused segments affects the occurrence of adjacent segment disease. Veeravagu et al. reported that the incidence of revision surgery is 3.4% per year for multilevel ACDF and 2.9% per year for single-level fusion. An increasing number of fused vertebrae correlates with increasing incidences of reoperation [12]. In contrast, another study showed that adjacent-segment disease is less common after multilevel fusion surgery because multilevel fusions usually include high-risk levels such as C5-C6 or C6-C7. In addition, multilevel fusions have an end adjacent to segments that are at lower risk for the development of new degeneration [13]. Ishihara et al. reported lower rates of clinically significant adjacent segment disease (ASD) in patients undergoing multilevel cervical arthrodesis. Furthermore, some studies report that the number of arthrodesis segments is not a significant risk factor for adjacent-segment disease [14].

## Conclusions

ACDF is an effective surgical option for treating degenerative disc disease across multiple levels. However, based on the literature, multilevel ACDF is a very challenging procedure because it can be associated with greater reoperation, complication, and pseudarthrosis rates when compared to single-level ACDF. The surgeons must recognize these factors and educate patients appropriately when deciding the appropriate procedure for cervical radiculopathy. We report the case of a patient who presented with complaints of progressively worsening weakness in the right upper and lower extremities who underwent successful five-level ACDF with substantial improvement in her clinical symptoms. We performed this procedure through a standard transverse incision from the midline to the medial border of the SCM within a pre-existing neck crease, showing that in select patients, extreme multilevel ACDF can be performed with proper anatomical dissection and without the need for multiple or longitudinal incisions.
